# Investigating the white matter correlates of reading performance: Evidence from Chinese children with reading difficulties

**DOI:** 10.1371/journal.pone.0248434

**Published:** 2021-03-11

**Authors:** Natalie Yu-Hsien Wang, Hsiao-Lan Sharon Wang, Yi-Chun Liu, Yi-Peng Eve Chang, Jun-Cheng Weng

**Affiliations:** 1 Department of Audiology and Speech-Language Pathology, Asia University, Taichung, Taiwan; 2 Department of Special Education, National Taiwan Normal University, Taipei, Taiwan; 3 Department of Radiology, Taichung Veterans General Hospital, Taichung, Taiwan; 4 Department of Counseling and Clinical Psychology, Columbia University, New York City, NY, United States of America; 5 Department of Medical Imaging and Radiological Sciences, Bachelor Program in Artificial Intelligence, Chang Gung University, Taoyuan, Taiwan; 6 Medical Imaging Research Center, Institute for Radiological Research, Chang Gung University and Chang Gung Memorial Hospital at Linkou, Taoyuan, Taiwan; 7 Department of Psychiatry, Chang Gung Memorial Hospital, Chiayi, Taiwan; University of California, San Francisco, UNITED STATES

## Abstract

**Purpose:**

Reading comprehension is closely associated with word recognition, particularly at the early stage of reading development. This association is reflected in children with reading difficulties (RD) who demonstrate poor reading comprehension along with delayed word recognition or reduced recognition accuracy. Although the neural mechanisms underlying reading comprehension and word recognition are well studied, few has investigated the white matter (WM) structures that the two processes potentially share.

**Methods:**

To explore the issue, behavioral scores (word recognition & reading comprehension) and diffusion spectrum imaging (DSI) were acquired from Chinese-speaking children with RD and their age-matched typically developing children. WM structures were measured with generalized fractional anisotropy and normalized quantitative anisotropy to optimize fiber tracking precision.

**Results:**

The children with RD performed significantly poorer than the typically developing children in both behavioral tasks. Between group differences of WM structure were found in the right superior temporal gyrus, the left medial frontal gyrus, the left medial frontal gyrus, and the left caudate body. A significant association between reading comprehension and Chinese character recognition and the DSI indices were found in the corpus callosum. The findings demonstrated the microstructural difference between children with and without reading difficulties go beyond the well-established reading network. Further, the association between the WM integrity of the corpus callosum and the behavioral scores reveals the involvement of the WM structure in both tasks.

**Conclusion:**

It suggests the two reading-related skills have partially overlapped neural mechanism. Associating the corpus callosum with the reading skills leads to the reconsideration of the right hemisphere role in the typical reading process and, potentially, how it compensates for children with reading difficulties.

## Introduction

Reduced reading comprehension is one of the characteristics commonly found among poor readers. According to the simple view of reading [1, 2], reading comprehension is supported by decoding (e.g. word recognition) and linguistic comprehension (e.g. phonological/morphological awareness). Evidence has demonstrated that both components are highly correlated with reading comprehension [3–5] and the combination of these components accounts for a large variance in reading comprehension [6, 7]. However, the relative contribution of these components varies across age. In the first few years of primary school, word recognition makes the greatest contribution to reading comprehension whilst linguistic comprehension contributes the least [8]. Therefore, the individual difference in word recognition should be the primary contributor to reading comprehension at this stage. Contrarily, starting from the fourth grade, the contribution of linguistic comprehension has increased while the contribution of word recognition decreased. Since word recognition is closely linked to reading comprehension at the early stage of reading development, this study focuses on the investigation of whether the association is reflected in white matter structures.

### Word recognition and reading comprehension

The research involved typically developing children and children with reading difficulties (RD) has provided cross-linguistic evidence in support of the simple view of reading [7, 9]. In comparison to age-matched typically developing children, poor word recognition performance is constantly reported among children with and/ or at risk of RD. For example, McBride-Chang et al. evaluated word recognition of three groups of Chinese preschoolers, including typically developing children, children with language delayed, and children with a family risk of RD [10]. McBride-Chang et al. reported that typically developing children had higher word recognition accuracy than the other two groups. In their study, word recognition was assessed with simple words that are suitable for preschoolers. Their result revealed that Chinese word recognition skill was a sensitive behavioral index for potential RD. Further, a cross-linguistic meta-analysis by Florit and Cain demonstrated that word decoding accuracy is a strong predictor of reading comprehension for English-speaking children and decoding fluency, which involved both speed and accuracy. It constantly predicted reading comprehension success in European children (e.g. Greek, Dutch, German, & Spanish) [9]. Research conducted with non-alphabetic language speakers, such as Chinese and Cantonese, demonstrated similar results. Further, word recognition accuracy, along with word-level linguistic comprehension, were the strong predictors of reading comprehension among Cantonese-speaking schooling children [11].

The contribution of word recognition to reading comprehension has been examined using regression analyses. For instance, Ho et al. assessed the decoding skills (word recognition accuracy & fluency) and linguistic comprehension (morphological awareness, vocabulary knowledge, morpho-syntactic skills, and discourse skills) performance of a group of Cantonese-speaking schooling children [7]. Their best fitting model accounted for 83% of the variance in reading comprehension, revealed that decoding and linguistic comprehension contributed 37% and 46% respectively. This result largely mirrored the evidence from populations of alphabetic languages [12–14]. Although the percentage of contribution of word recognition to reading comprehension varies depends on how the abilities were assessed [15], word recognition constantly accounts for above thirty-percent of variance among children around age ten. Therefore, it may be considered as a stable predictor of reading comprehension at the early stage of reading development.

### White matter tracts associated with reading

A reading network established based on functional magnetic resonance imaging (MRI) evidence includes the left tempo-parietal region, the left inferior frontal gyrus, and the left ventral occipitotemporal region [16–18]. To successfully complete a reading task, multiple regions within the reading network are co-activated. The communications between the cortical regions are therefore important and rely on the underlying white matter (WM) structures. The left arcuate fasciculus is assumed to play a crucial role as it connects two major regions in the network, the left tempo-parietal region, and the left inferior frontal gyrus. The other part of the network, the left ventral occipitotemporal regions, is connected with the left inferior frontal gyrus through the left inferior fronto-occipital fasciculus. Drawing evidence from children with RD, many studies have suggested that poor reading proficiency is associated with reduced fractional anisotropy (FA), which indexes the WM integrity, in the left tempo-parietal and frontal areas, such as the left arcuate fasciculus [19–21] and corona radiate [22]. Some studies also reported association between reading skills and the inferior longitudinal fasciculus [23], or the inferior fronto-occipital fasciculus [16]. The discrepancy across studies is likely to result from differences in the participant age range, the measure used to evaluate reading related skills, or the domains of reading skills evaluated (e.g. word recognition, word/sentence level of comprehension, & phonological awareness). For instance, Deutsch, Dougherty, Bammer, Siok, Gabrili, and Wandell [24] examined the relation of FA with word recognition, rapid automatized naming, and phonological awareness respectively. Positive correlations were found between FA and word recognition as well as rapid automatized naming in the left temporoparietal region; yet, the FA value of this was not correlated with participants’ phonological awareness.

On the other hand, the interhemispheric deficit theory proposed that poor reading is a result of potential abnormal communication between the hemispheres [25, 26]. The verification of this theory involves investigation of the structural concomitant of callosal dysfunction in RD population. Fine, Semrud-Clikeman, Keith, Stapleton, and Hynd George [27] provided supporting evidence for the theory by reporting reduced FA values within the midbody of the corpus callosum in both children and adults with RD. The midbody of the corpus callosum is implicated in processing primary sensory information. In the process of reading, rapid sensory integration is essential for grapheme-to-phoneme mapping. Reduced integrity of the midbody of the corpus callosum may result in inefficient processing and ultimately disrupt reading fluency. A few studies have also drawn attention to the potential association between various reading related skills and the integrity of corpus callosum [28, 29]. Hasan et al. found that the average FA in the callosal microstructure was significantly greater in children with word recognition problem, compared to typically developing children [29]. Further, the FA value in the posterior midbody of the corpus callosum correlated negatively with word recognition performance. In addition to children with word recognition problem, the study also involved a group of children with reading comprehension problem who performed below average on a passage reading task. Hasan et al. reported no difference in the callosal microstructure between children with reading comprehension problem and typically developing children. This result is considered as counter evidence for the interhemispheric deficit theory. Moreover, it shows the lack of inconsistency in the existing literature and the further investigation is required to identify if the WM associates of the two reading related skills potentially overlapped.

### Aims of the study

Behavioral evidence has demonstrated that word recognition is a strong predictor of reading comprehension in the early stage of reading development. This study aims to firstly verify how reading performance may be reflected by WM. Further, the investigation also aims to explore whether the two closely related processes are supported by shared WM correlates. To achieve this, we adopted two WM indices generated from diffusion spectrum imaging (DSI), which provides better reconstruction of nerve fibers than the widely used diffusion tensor imaging (DTI).

The majority of DTI studies use FA value to describes the degree of anisotropy of a diffusion process. Yet, FA has received criticism regarding its accuracy in presenting the diffusion process [30]. By contrast, DSI is a unique q-space reconstruction method derived from q-space imaging. When compared to DTI, DSI could be applied to a wide range of q-space datasets for a more accurate and sophisticated diffusion MR approach. DSI was used to overcome limitations including, but not limited to the resolution of complicated neural structures (e.g., fiber crossings) and the detection of structural changes in both white and gray matter. Therefore, the potential drawback of FA can be improved with a higher order orientation-distribution-function-based model, which defines generalized fractional anisotropy (GFA) to provide more accurate estimates of the anisotropy. Another orientation-distribution-function-based index, quantitative anisotropy (QA), is found to be less sensitive to the partial volume effects of crossing fibers and free water, resulting in better resolution than FA for WM fiber tracking.

## Methodology

### Participants

Two groups of children were recruited from the same primary school for the study, including twenty-four children with reading difficulties [RD (mean age = 113.95 months; *SD* = 10.00)] and twenty-two typically developing children [controls (mean age = 114.25 months; *SD* = 11.52)]. The RD individuals were screened from the databased of the Special Education Division, Department of Education of Taipei City Government. The participants with RD were two-year behand typically developing children in terms of reading skills, assessed by the Chinese Character Recognition Test [31]. The participants with RD must have no other neurological or psychiatric disorder.

The intelligence quotient (IQ) of all participants were assessed with the Abbreviated Wechsler Intelligence Scale for Children–Fourth Edition (WISC-IV) [32] to ensure normal IQ (full scale > 80). All the participants took part in two sessions. In one session, they completed two behavioral tasks (reading comprehension & Chinese character recognition); in the other session, DSI data was acquired. Prior to the participation, the participants and their parents to debriefed to ensure that they had understood the study and a written informed consent was signed. The study was approved by the Research Ethics Committee at National Taiwan University (NTU-REC No. 201310EM016).

### Behavioral tasks

The Reading Comprehension Screening Test [33], a standardized assessment tool, was used to evaluate participants’ reading comprehension. The reading material involves sentences and short texts. After reading, the participants were to either name choose an appropriate topic for the reading, identify synonyms, or reason and interpret the text. Overall, the test consisted of twenty-seven questions and each correct response was given. The cut-off score for children at age nine is 12. The test-retest reliability coefficient of this test is .87.

The performance on Chinese character recognition was measured with the Graded Chinese Character Recognition Test [31], which is a widely used standardized tool for identifying reading difficulties. The test involved reading aloud a list of 200 Chinese characters with progressively lowered printed-word-frequency. All characters were real Chinese characters and semantically unrelated to one another. This test generates a recognition accuracy score, the maximum of which is 200, and provides error type analysis that reflects potential decoding failure leading to inaccurate recognition. The cut-off scores for the third and fourth graders are 49 and 65 respectively. The test score is positively correlated (*r* value between .36 and .69; *p* values between .05 and .01) with children’s Chinese language performance in school. The split-test reliability coefficient of this test is .99.

### Diffusion imaging acquisition

The diffusion images were acquired on a 3T MRI system (Prisma, Siemens, Germany). The scanning parameters were as follows: repetition time = 6700 ms, echo time = 97 ms, FOV = 222 x 222 mm^2^, matrix size = 82 x 82, in-plane resolution = 2.7 x 2.7 mm^2^, slice thickness = 2.7 mm, and number of slice = 50. A total of 128 diffusion orientations was sampled in the half q-space with b-values from 0 to 5000 s/mm^2^ (see S1 Table). The duration of the scan was around fourteen minutes for each participant.

### DSI data pre-processing and analysis

Prior to pre-processing, the diffusion images of all participants were checked for potential motion artifacts to ensure the accuracy of the results. For each participant, the original diffusion images were processed with eddy current and susceptibility-induced phase distortion correction using FSL (FMRIB Software Library, Oxford, UK). Large FOV and fat saturation sequence were also adopted to avoid wrap-around of the fat saturation artifact. The corrected diffusion images were registered to the b0 (null) image in native diffusion space using a linear transformation. The registered images were spatially normalized to the standard T2 weighted template, Montreal Neurological Institute (MNI) template, after an affine transformation with 12 degrees of freedom and non-linear warps using Statistical Parametric Mapping 8 (SPM8, Wellcome Department of Cognitive Neurology, London, UK). For the DSI analysis, DSI reconstruction was performed using DSI Studio (National Taiwan University, Taipei, Taiwan) and the GFA and normalized quantitative anisotropy (NQA) mapping were calculated [34].

To verify DSI indices of the RD group were potentially different from the controls, a two sample *t*-test was performed using SPM. The result was visualized using xjView toolbox (https://www.alivelearn.net/xjview). A false discovery rate (FDR)-corrected *p*-value of less than 0.05 and cluster size more than 200 was considered statistically significant. Further, four multiple regression analyses, with sex and IQ as covariates, were applied to identify potential associations between the two behavioral measures (reading comprehension & Chinese character recognition) and the DSI indices (GFA & NQA) respectively. The multiple analyses were performed using SPM. We correlated brain diffusion indices of each brain voxel across each individual with the behavioral scores using general linear model (GLM). It is simply the entering of covariates (e.g. word recognition or reading comprehension), which can be tested for correlations with the brain diffusion indices (e.g. GFA or NQA) change across subjects. Because anisotropy was restricted to white matter, we used ICBM template to identified tracts with statistically significant effects.

## Results

Descriptive data of behavioral measures are presented in Table 1, which contains the full-scale IQ, a composite of the four indices, the reading comprehension score, and Chinese character recognition accuracy. Two sample *t*-tests were applied to compare the performance of the two groups on all behavioral measures. Despite that the participants had normal IQ, the full-scale IQ of the RD group was significantly lower than that of the control group (*t* (46) = 4.567, *p* < .001; *d* = 1.45). However, it should be noted that, as demonstrated in Table 1, the reduced performance was found in tasks (similarities & digit span) involved linguistic knowledge. The RD group also demonstrated significantly poorer reading comprehension (*t* (46) = 8.700, *p* < .001; *d* = 2.56) and Chinese character recognition (*t* (46) = 4.042, *p* < .001; *d* = 1.33) than the control group.

**Table 1:** The mean and standard deviation, shown in brackets, of the behavioral measures and group comparison results.

	controls	RD	two sample t-test (*t*)
WISC-IV (estimated full scale IQ)	105.12 (5.62)	92.64 (10.61)	4.567**
Similarities	11.80 (2.21)	8.38 (2.78)	3.946**
Digit span	11.87 (2.67)	8.95 (3.09)	2.948*
Matrix reasoning	9.40 (3.31)	8.38 (2.69)	1.017
Symbol search	10.07 (2.40)	9.71 (2.17)	0.459
Reading comprehension	21.23 (4.34)	10.13 (4.31)	8.700**
Chinese character recognition	92.82 (23.43)	62.38 (22.16)	4.042**

*p < .05

**p < .01

**Fig 1:**
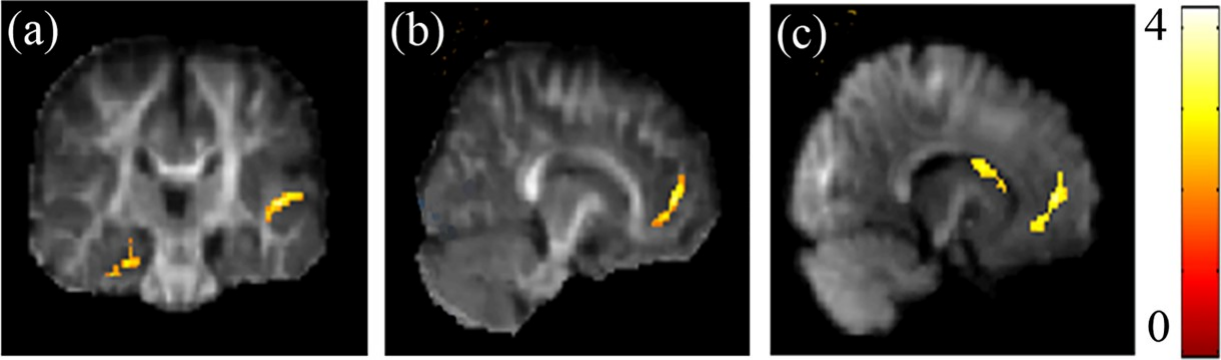
Group effect on DSI indices. Significantly (a) reduced GFA in the right superior temporal gyrus and (b) the left medial frontal gyrus, (c) reduced NQA in the left medial frontal gyrus and the left caudate body were found in RD compared with controls. The color bar presents *t*-score (corrected *p* < 0.05).

Results of two sample *t*-test also revealed altered DSI indices among children with RD. Compared to the controls, the RD group had significantly lower value of GFA in the right superior temporal gyrus, Fig 1(A), and the left medial frontal gyrus, Fig 1(B). Also, the RD group demonstrated reduced NQA values in left medial frontal gyrus and the left caudate body, Fig 1(C).

**Fig 2:**
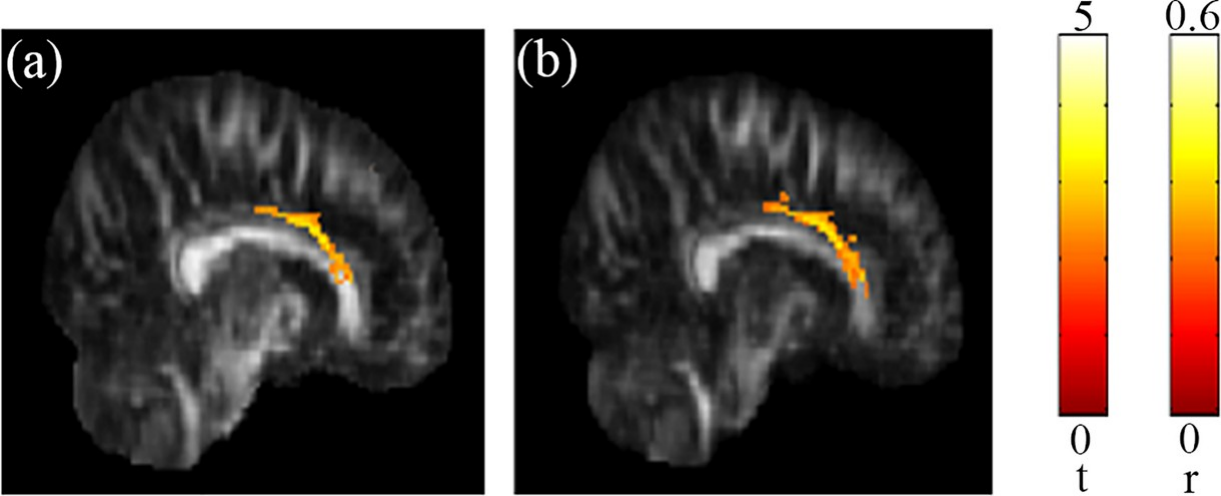
Association between the reading comprehension score and diffusion indices. Positive correlations between the reading comprehension score and (a) GFA and (b) NQA values were found in the corpus callosum. The color bar presents *t*-score and *r* (corrected *p* < 0.05).

**Fig 3:**
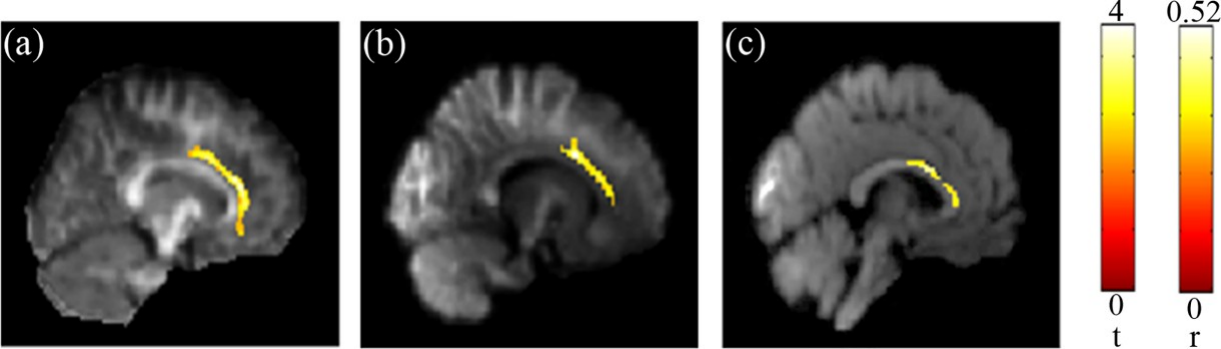
Association between the Chinese character recognition accuracy and diffusion indices. Positive correlations between the Chinese character recognition accuracy and the DSI indices (GFA & NQA) were found in the cingulate gyrus (a & b) and (c) the corpus callosum. The color bar presents *t*-score and *r* (corrected *p* < 0.05).

**Fig 4:**
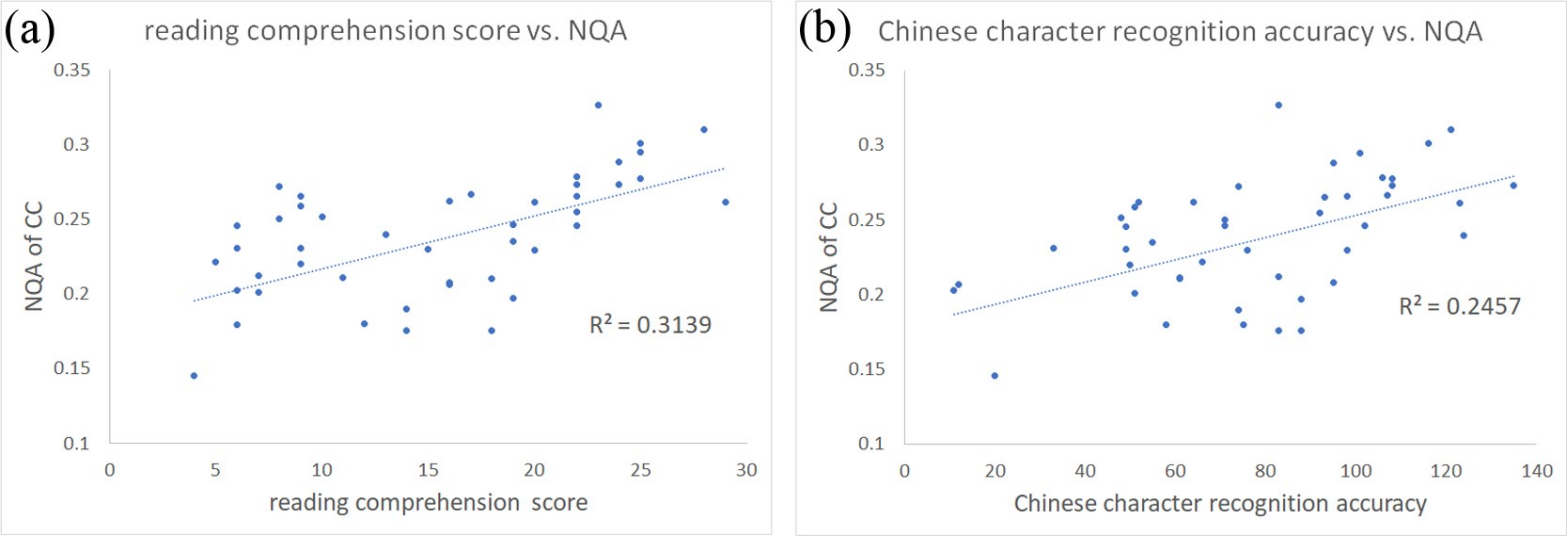
The scatter plot of the reading comprehension score/Chinese character recognition accuracy and diffusion indices. (a) The scatter plot showing all individuals’ reading comprehension score and NQA from the corpus callosum region. (b) The scatter plot showing all individuals’ Chinese character recognition accuracy and NQA from the corpus callosum region.

The results of the multiple regression analysis showed significant association between the performance on reading comprehension and the GFA and NQA in the corpus callosum (Fig 2). That is, the values of both indices in corpus callosum increased along with the reading comprehension score. On the other hand, Chinese character recognition accuracy was significantly associated with both GFA and NQA values in the corpus callosum as well as the cingulate gyrus (Fig 3). Higher values were found to be linked with higher recognition score. The detail of the significant results (corrected *p* < 0.05) mentioned above were also listed in Table 2. The small clusters that reached significance (*p* < 0.05) but without correction in other areas of the brain were not shown, but also listed as Table 3. The scatter plot of all individuals’ reading comprehension score vs. NQA from the corpus callosum region can be found in Fig 4A, and the scatter plot of all individuals’ Chinese character recognition accuracy vs. NQA from the corpus callosum region can be also found in Fig 4B.

**Table 2:** The small clusters that reached significance with FDR correction.

***t*-test (Control > RD)**			
White matter structures	Coordinates (X, Y, Z)	Cluster size	*Corrected p-*value
Right superior temporal gyrus	44.6, -27.69, 3.13	200	0.05
Left medial frontal gyrus	-13.58, 38.19, -3.33	200	0.05
Left caudate	-13.57, 5.9, 19.94	200	0.05
**multiple regression analysis of reading comprehension and DSI indices**			
White matter structures	Coordinates (X, Y, Z)	Cluster size	*Corrected p-*value
Corpus callosum	-10, 26, 12	400	0.02
**multiple regression analysis of Chinese character recognition accuracy and DSI indices**			
White matter structures	Coordinates (X, Y, Z)	Cluster size	*Corrected p-*value
Corpus callosum	0, 24, 10	600	0.01
Right Cingulate gyrus	12.28, 2.02, 35.45	600	0.01

**Table 3:** The small clusters that reached significance (*p* < 0.05) without FDR correction.

***t*-test (Control > RD)**			
White matter structures	Coordinates (X, Y, Z)	Cluster size	*P-*value
Left fusiform gyrus	-25.21, -41.9, -17.55	200	0.04
Middle frontal gyrus	-21.33, -9.6, 47.09	100	0.02
Cingulate gyrus	-12.28, -9.6, 32.87	100	0.02
**multiple regression analysis of reading comprehension and DSI indices**			
White matter structures	Coordinates (X, Y, Z)	Cluster size	*P-*value
Right superior frontal gyrus	25.21, -5.73, 70.36	150	0.02
Right caudate	16, 2.02, 14.77	150	0.02
Left superior parietal gyrus	-22, -56, 52	130	0.05
**multiple regression analysis of Chinese character recognition accuracy and DSI indices**			
White matter structures	Coordinates (X, Y, Z)	Cluster size	*P-*value
Right precuneus	-21.33, -71.6, 31.58	150	0.01
Left inferior parietal gyrus	-32.97, -54.81, 43.21	100	0.05
Right caudate	16, 2.02, 14.77	150	0.01

## Discussion

This study set out to investigate the association between Chinese word recognition and reading comprehension at the early stage of reading development and how this relationship might reflect on WM structures. Reading skills were measured with standardized assessment tools. The DSI data was analyzed with two types of fiber tracking technique to generate GFA and NQA respectively. As demonstrated in the result section, the GFA and the NQA values were largely in agreement with each other in terms of the regions identified for the between group differences and the association with behavioral scores. However, in addition to the left medial frontal gyrus, lower GFA was found in the right superior temporal gyrus whilst lower NQA was in the left caudate body. The evidence provided by this study largely supports the existing neurological theories established based on fMRI and the FA index of DTI. These discrepancies suggest that different fiber tracking techniques lead to different findings within the reading network.

### Atypical structures in children with reading difficulties

The between group difference in WM structure in the left medial frontal gyrus echoes the existing evidence showing disrupted functional connectivity in children with RD [35, 36]. This finding is different from the commonly reported evidence suggesting reduced connectivity within the reading network, such as lower FA values in the left arcuate fasciculus and left temporo-parietal white matter. However, cross-linguistic evidence has associated the left medial frontal gyrus with visual word processing [37] as well as phonological processing of visually presented words [38–41]. That is, the left medial frontal gyrus plays an important role in mapping orthography to phonology. According to Tan et al. [41] the left medial frontal gyrus, along with the left inferior frontal gyrus, was activated in Chinese speakers when performing a visually presented homophone judgement task. Similarly, in a series of fMRI studies by Cao and colleagues [39, 40], atypical activations of the left medial frontal gyrus as well as the left inferior frontal gyrus had been observed in English-speaking children with RD during the performance of visual word rhyming judgment task. Compared to typically developing children, the children with RD demonstrated reduced activation in these regions. The level of activation positively correlated with judgement accuracy. Hence, the reduced integrity of WM found in the left medial frontal gyrus may account for the atypical activation previously reported. It should be noted that we should not consider this finding as direct evidence of impaired orthography to phonology mapping in our participant group due to the fact that other WM structures underlies the lexical processing network were not significantly different between groups.

In addition, the medial frontal gyrus is associated to the control of attention and executive function. Many studies have reported different domains of attentional deficits, including auditory and visuo-spatial attention [42, 43], as well as reduced inhibitory control of executive function [44] among children with RD. Indeed, these aspects of cognitive functions are inseparable from reading performance. In the early stage of reading, inhibitory control involves in the process of orthography to phonology mapping, during which the reader must inhibit the competing phonological code to retrieve the target sound for an orthography. At sentence level, inhibition of competing semantic information is required for successful comprehension. However, the current evidence cannot verify whether the left medial frontal gyrus is linked to the non-linguistic cognitive factors might be found among children with RD because attention and executive function assessments had not been included in this study. Further research is required to verify the issue.

### White matter associating with reading skills

This study reports that word recognition and reading comprehension performance were significantly associated with the integrity of the corpus callosum. This finding supports our perdition that the two closely linked reading abilities might be associated with common WM structures. Further, it should be noted that when behavioral scores of the reading tasks (word recognition & comprehension) were not considered, there was no between group difference in the callosal microstructure. This finding argues against studies, e.g. [29], suggesting that the corpus callosum could be a potential biomarker for dyslexia.

The associations between the corpus callosum and word recognition and reading comprehension reported in this study may be considered as supporting evidence for the interhemispheric deficit theory. Moreover, these findings lead to the reconsideration of the role of right hemisphere in reading. The role of the right hemisphere in word recognition has been discussed in a few functional magnetic resonance imaging, [41, 45, 46], and DTI studies [47, 48]. Tan et al. pointed out that, in typically developing readers, Chinese word recognition extensively activated the left frontal and temporal cortices and the right lateralization of visual system (BAs 17–19) [45]. Moreover, compared to reading English, more right hemisphere cortical regions, such as BAs 47/45, 7, 40/39, were involved [41]. One possible account for the cross-linguistic difference is that logographic script required elaborated visual analysis of its spatial information.

Furthermore, reduced DSI index values in the corpus callosum leads to disrupted interhemispheric communication. It is likely that impaired callosal structure would be less efficient at transferring linguistic information to the reading network in the left hemisphere causing the brain to be less lateralized. Children and adults with RD, therefore, recruit the right hemisphere homologs during reading and reading-related tasks as a compensatory mechanism [49, 50]. Despite the results of this study do not directly reflect the functional evidence, they shade lights on the importance of interhemispheric connectivity in achieve successful reading at both word and passage level. This study contributes to the further understanding of two important reading skills and their white matter correlates.

## Conclusion

This study provides further understanding of the WM structure in children with and without RD using advanced DSI techniques. The main contribution is showing that reduced integrity of corpus callosum is associated with low level reading skill, decoding, as well as reading comprehension, which is considered as a higher level reading ability that require more cognitive effort. The corpus callosum is not part of the well-established reading network but its role in interhemispheric connection implies the potential importance of the right hemisphere for reading-related task performance. Yet, the corpus callosum should not be considered as a neural marker for RD due to the findings are task-specific. The study has a couple of limitations. One is that the difference between children with and without RD found in the left medial frontal gyrus should not be overtly generalized and regarded as a neural marker, larger sample size is needed to establish more reliable result. The other is the lack of functional imaging data for multimodality to provide direct evidence associating the WM structures with potential functional deficits in children with RD.

## Supporting information

S1 Table The b-values for the 128 diffusion directions and null. (DOCX)
