# Supplementary material for: Investigating the white matter correlates of reading performance: Evidence from Chinese children with reading difficulties
Source: PLoS One. 2021 Mar 11;16(3):e0248434. doi: 10.1371/journal.pone.0248434 (PMC7951916; doi:10.1371/journal.pone.0248434)
Supplement: S1 Table — (DOCX) [file pone.0248434.s001.docx]

S1 Table. The b-values for the 128 diffusion directions and null.

| No. | b-value | x | y | z |
| --- | --- | --- | --- | --- |
| 0 | 0 | 0 | 0 | 0 |
| 1 | 300 | -0.104197 | 0.984637 | 0.14012 |
| 2 | 300 | -0.0015151 | 0.150264 | -0.988645 |
| 3 | 300 | 0.994207 | 0.106872 | 0.0114783 |
| 4 | 650 | -0.77592 | 0.624805 | 0.0869839 |
| 5 | 600 | -0.702064 | 0.0338131 | -0.711311 |
| 6 | 650 | -0.0713816 | 0.801844 | -0.593255 |
| 7 | 650 | 0.0741655 | -0.593378 | -0.8015 |
| 8 | 650 | 0.628327 | 0.76992 | 0.11148 |
| 9 | 600 | 0.705007 | 0.179134 | -0.686204 |
| 10 | 950 | -0.632238 | 0.596099 | -0.494916 |
| 11 | 950 | -0.5125 | -0.543976 | -0.664405 |
| 12 | 950 | 0.514146 | 0.714331 | -0.474747 |
| 13 | 950 | 0.6355 | -0.425341 | -0.644379 |
| 14 | 1250 | -0.103412 | 0.98415 | 0.144066 |
| 15 | 1250 | 0.00105314 | 0.148928 | -0.988847 |
| 16 | 1250 | 0.994387 | 0.104708 | 0.0152062 |
| 17 | 1550 | -0.935611 | 0.349765 | 0.0479107 |
| 18 | 1550 | -0.888961 | -0.0275869 | -0.457152 |
| 19 | 1550 | -0.537118 | 0.83478 | 0.121025 |
| 20 | 1550 | -0.442779 | 0.0873772 | -0.892363 |
| 21 | 1550 | -0.0915414 | 0.946435 | -0.309645 |
| 22 | 1550 | -0.0443996 | 0.573318 | -0.818129 |
| 23 | 1550 | 0.0479699 | -0.309457 | -0.949703 |
| 24 | 1550 | 0.0933448 | -0.815114 | -0.57173 |
| 25 | 1550 | 0.350734 | 0.926473 | 0.136506 |
| 26 | 1550 | 0.446318 | 0.179239 | -0.87674 |
| 27 | 1550 | 0.842263 | 0.533263 | 0.0788916 |
| 28 | 1550 | 0.890531 | 0.159781 | -0.42594 |
| 29 | 1850 | -0.85343 | 0.379412 | -0.357357 |
| 30 | 1850 | -0.768478 | -0.426738 | -0.476798 |
| 31 | 1900 | -0.489539 | 0.822399 | -0.289849 |
| 32 | 1850 | -0.44651 | 0.481578 | -0.754129 |
| 33 | 1850 | -0.361633 | -0.324475 | -0.874035 |
| 34 | 1900 | -0.319483 | -0.785948 | -0.529356 |
| 35 | 1900 | 0.320966 | 0.906079 | -0.275685 |
| 36 | 1850 | 0.364566 | 0.565348 | -0.739915 |
| 37 | 1850 | 0.449997 | -0.240736 | -0.85997 |
| 38 | 1900 | 0.491634 | -0.702242 | -0.514929 |
| 39 | 1850 | 0.769711 | 0.547248 | -0.328734 |
| 40 | 1850 | 0.855345 | -0.259056 | -0.448637 |
| 41 | 2500 | -0.77631 | 0.623936 | 0.0897054 |
| 42 | 2500 | -0.701946 | 0.0326921 | -0.711479 |
| 43 | 2500 | -0.0718045 | 0.800729 | -0.594707 |
| 44 | 2500 | 0.0746538 | -0.592521 | -0.802089 |
| 45 | 2500 | 0.628792 | 0.769139 | 0.114217 |
| 46 | 2500 | 0.704675 | 0.178313 | -0.686759 |
| 47 | 2800 | -0.731256 | 0.637459 | -0.242715 |
| 48 | 2800 | -0.696244 | 0.358908 | -0.621634 |
| 49 | 2800 | -0.626867 | -0.299286 | -0.719351 |
| 50 | 2800 | -0.592319 | -0.676209 | -0.438063 |
| 51 | 2800 | -0.399186 | 0.720857 | -0.566582 |
| 52 | 2800 | -0.260332 | -0.592883 | -0.762048 |
| 53 | 2800 | -0.103855 | 0.984014 | 0.144672 |
| 54 | 2800 | 0.00155214 | 0.148448 | -0.988919 |
| 55 | 2800 | 0.262804 | 0.789237 | -0.555013 |
| 56 | 2800 | 0.402006 | -0.524544 | -0.750496 |
| 57 | 2800 | 0.59359 | 0.774267 | -0.21946 |
| 58 | 2800 | 0.629326 | 0.495992 | -0.598282 |
| 59 | 2800 | 0.69913 | -0.162295 | -0.696332 |
| 60 | 2800 | 0.73279 | -0.539384 | -0.41483 |
| 61 | 2800 | 0.994443 | 0.10409 | 0.015745 |
| 62 | 3100 | -0.976258 | 0.214757 | 0.0282795 |
| 63 | 3100 | -0.942995 | -0.0519992 | -0.32872 |
| 64 | 3150 | -0.412753 | 0.901262 | 0.131764 |
| 65 | 3100 | -0.312212 | 0.108438 | -0.943803 |
| 66 | 3150 | -0.0978571 | 0.980064 | -0.172911 |
| 67 | 3100 | -0.0305507 | 0.452031 | -0.891479 |
| 68 | 3100 | 0.0343523 | -0.172153 | -0.984471 |
| 69 | 3150 | 0.0991344 | -0.88716 | -0.450688 |
| 70 | 3150 | 0.215145 | 0.966099 | 0.14271 |
| 71 | 3100 | 0.316163 | 0.173357 | -0.932732 |
| 72 | 3100 | 0.910206 | 0.409614 | 0.0611744 |
| 73 | 3100 | 0.944156 | 0.145373 | -0.295696 |
| 74 | 3450 | -0.930306 | 0.249357 | -0.268982 |
| 75 | 3450 | -0.867382 | -0.346258 | -0.357427 |
| 76 | 3450 | -0.392795 | 0.903752 | -0.170131 |
| 77 | 3450 | -0.329051 | 0.400113 | -0.855357 |
| 78 | 3450 | -0.266353 | -0.195313 | -0.94388 |
| 79 | 3450 | -0.20458 | -0.876896 | -0.434973 |
| 80 | 3450 | 0.205598 | 0.965539 | -0.159572 |
| 81 | 3450 | 0.269922 | 0.461943 | -0.844838 |
| 82 | 3450 | 0.332883 | -0.133206 | -0.933512 |
| 83 | 3450 | 0.394204 | -0.815066 | -0.424583 |
| 84 | 3450 | 0.868458 | 0.435061 | -0.237704 |
| 85 | 3450 | 0.93165 | -0.160186 | -0.326142 |
| 86 | 3750 | -0.632804 | 0.594719 | -0.495851 |
| 87 | 3750 | -0.512518 | -0.543207 | -0.66502 |
| 88 | 3750 | 0.514738 | 0.713283 | -0.475681 |
| 89 | 3750 | 0.635285 | -0.424637 | -0.645056 |
| 90 | 4050 | -0.884977 | 0.461011 | 0.0654635 |
| 91 | 4050 | -0.8266 | -0.004705 | -0.56277 |
| 92 | 4050 | -0.63783 | 0.762157 | 0.110858 |
| 93 | 4050 | -0.550014 | 0.0665519 | -0.8325 |
| 94 | 4050 | -0.0853069 | 0.900719 | -0.425945 |
| 95 | 4050 | -0.0556786 | 0.669192 | -0.741001 |
| 96 | 4050 | 0.0590258 | -0.42394 | -0.903765 |
| 97 | 4050 | 0.0875528 | -0.737516 | -0.669631 |
| 98 | 4050 | 0.464335 | 0.876054 | 0.130085 |
| 99 | 4050 | 0.553267 | 0.180691 | -0.813171 |
| 100 | 4050 | 0.769182 | 0.632029 | 0.0943312 |
| 101 | 4050 | 0.8287 | 0.168352 | -0.533773 |
| 102 | 4350 | -0.85222 | 0.483784 | -0.199186 |
| 103 | 4350 | -0.824115 | 0.260391 | -0.503022 |
| 104 | 4350 | -0.76848 | -0.267541 | -0.581258 |
| 105 | 4350 | -0.740806 | -0.569669 | -0.355927 |
| 106 | 4400 | -0.614043 | 0.7738 | -0.155514 |
| 107 | 4350 | -0.557728 | 0.327125 | -0.762843 |
| 108 | 4350 | -0.502002 | -0.200585 | -0.841285 |
| 109 | 4400 | -0.447078 | -0.804899 | -0.390202 |
| 110 | 4400 | -0.347751 | 0.840658 | -0.415166 |
| 111 | 4350 | -0.319508 | 0.6175 | -0.718755 |
| 112 | 4350 | -0.208234 | -0.435951 | -0.875549 |
| 113 | 4350 | -0.18083 | -0.738098 | -0.650009 |
| 114 | 4400 | 0.182832 | 0.895503 | -0.405768 |
| 115 | 4350 | 0.211289 | 0.67236 | -0.709428 |
| 116 | 4350 | 0.322896 | -0.381114 | -0.866309 |
| 117 | 4350 | 0.350028 | -0.683352 | -0.640711 |
| 118 | 4400 | 0.447965 | 0.88351 | -0.136884 |
| 119 | 4350 | 0.505163 | 0.437035 | -0.744185 |
| 120 | 4350 | 0.561011 | -0.0906311 | -0.822832 |
| 121 | 4350 | 0.615223 | -0.695198 | -0.371753 |
| 122 | 4350 | 0.741766 | 0.64838 | -0.171426 |
| 123 | 4350 | 0.770473 | 0.425074 | -0.475062 |
| 124 | 4350 | 0.826476 | -0.102582 | -0.553548 |
| 125 | 4350 | 0.853471 | -0.404892 | -0.328099 |
| 126 | 5000 | -0.103811 | 0.983964 | 0.145045 |
| 127 | 5000 | 0.0017351 | 0.148201 | -0.988956 |
| 128 | 5000 | 0.994471 | 0.103742 | 0.0162782 |
